# Supplementary figures and images for: Phylogenetics and genomic variation of Hepatocystis isolated from shotgun sequencing of wild primate hosts
Source: PLoS Pathog. 2025 Jun 18;21(6):e1013240. doi: 10.1371/journal.ppat.1013240 (PMC12187015; doi:10.1371/journal.ppat.1013240)

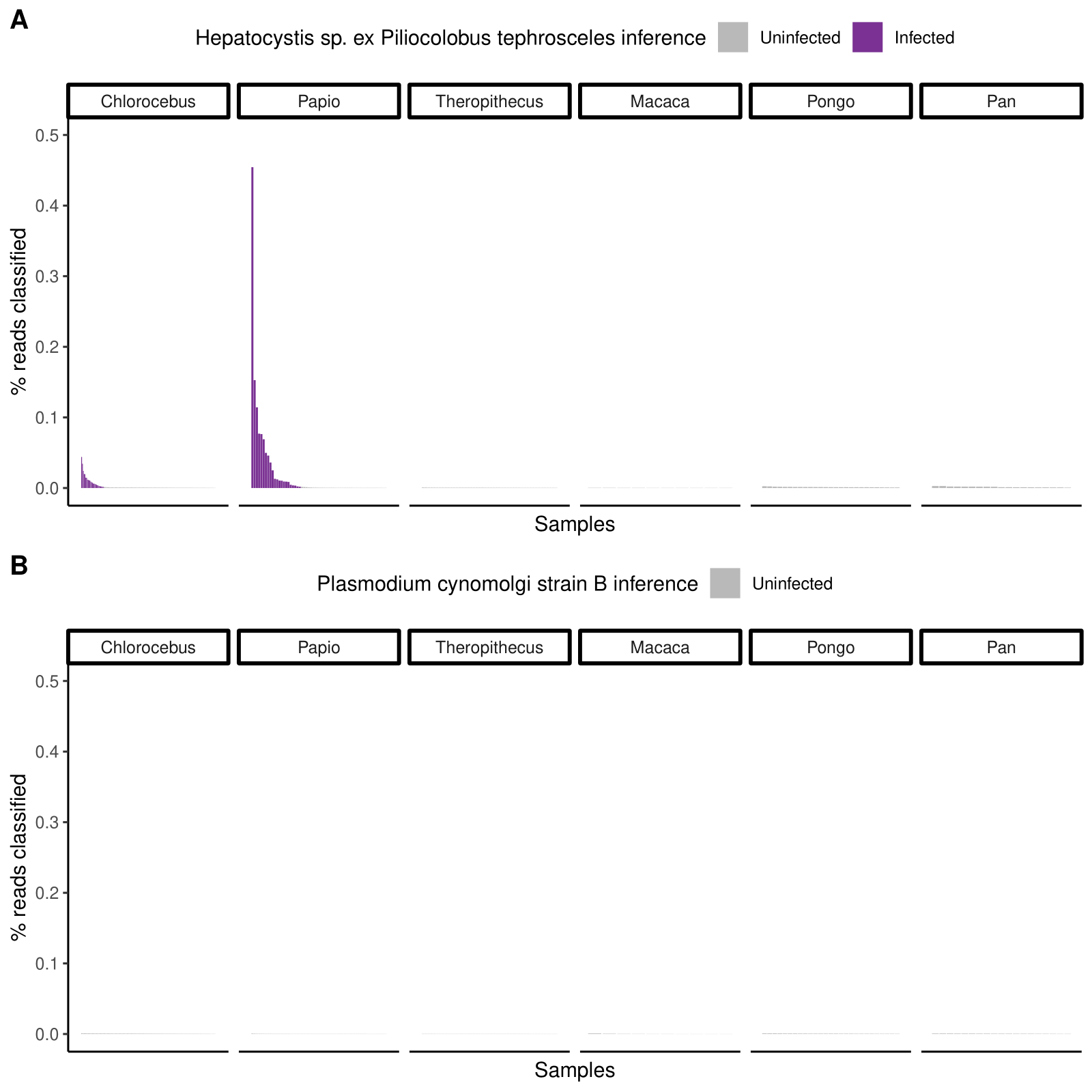

Supplement: S1 Fig — Samples inferred as infected are colored purple, while those considered uninfected are shown in gray. Samples are grouped by host genus. (TIF) [file ppat.1013240.s001.tif]

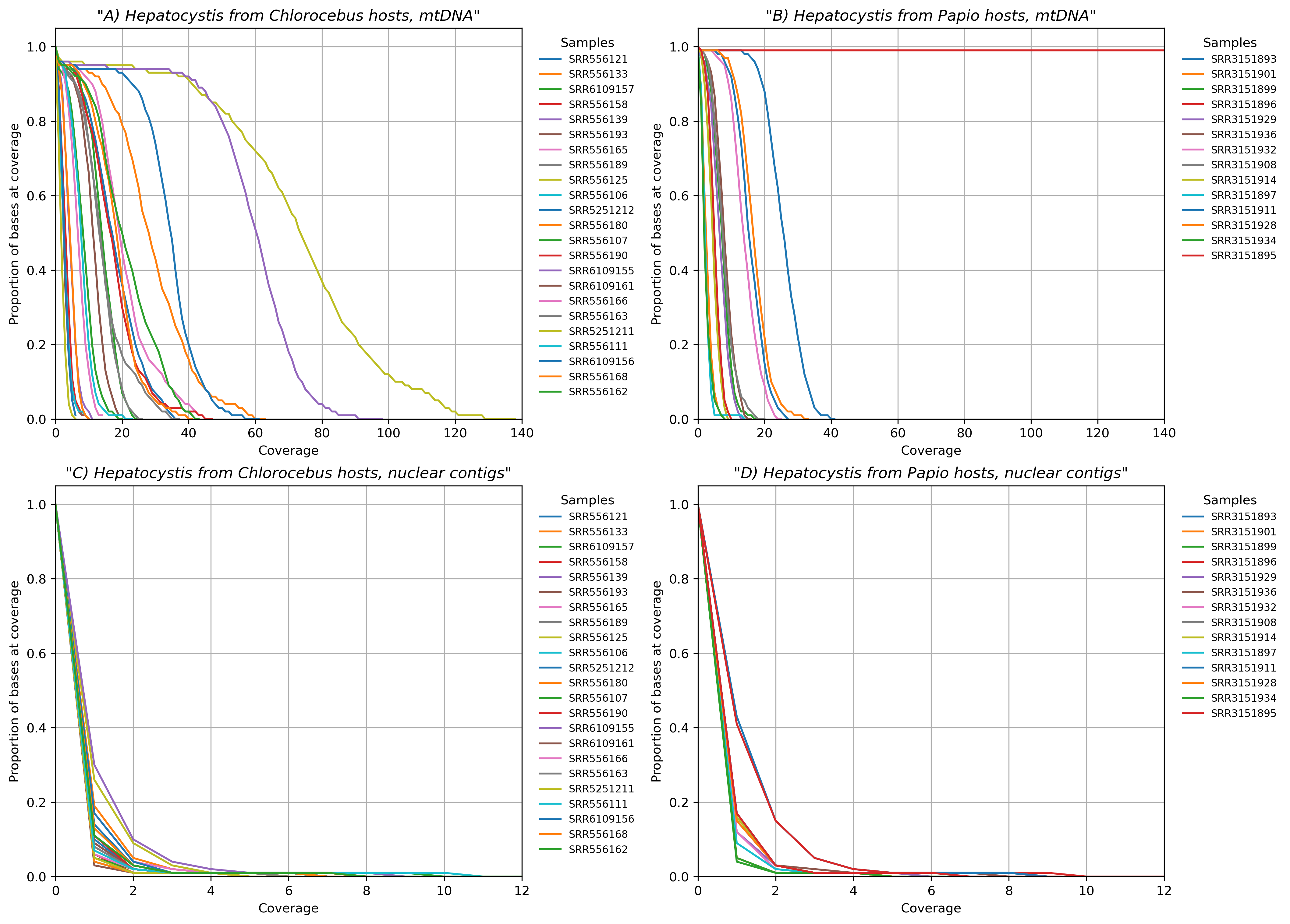

Supplement: S2 Fig — (A) mtDNA contig in cHep. (B) mtDNA in pHep. (C) nuclear contigs in cHep. (D) Nuclear contigs in pHep. (TIF) [file ppat.1013240.s002.tif]

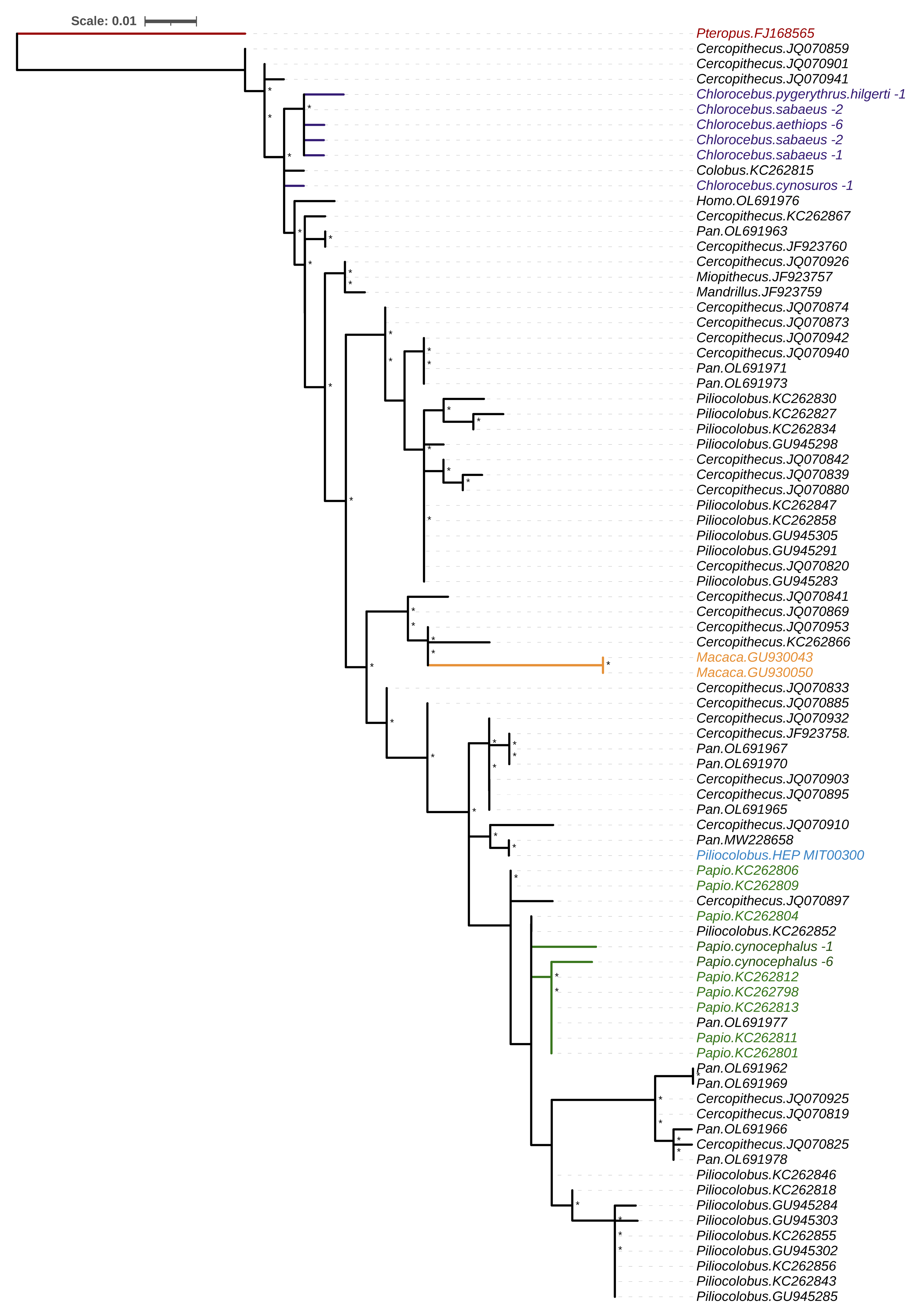

Supplement: S3 Fig — The maximum likelihood tree was constructed with IQ-TREE from the 20 cytb sequence assemblies in our dataset together with the Hepatocystis reference sequence and additional 75 unique publicly available Hepatocystis sequences from various NHPs sequenced via PCR (alignment length = 287 bp, minimum sequence length = 287 bp, sites present in all sequences = 287 bp). Tips representing sequences from our dataset are colored and labeled with the host species name and number of samples carrying the same sequence. Tips from published studies are labeled by host genus and a sample ID. Bootstrap values >50% are indicated with an asterisk. Color key: red – outgroup from bat host. Purple – Chlorocebus hosts from our study. Dark green – Papio hosts from this study. Light green – Papio hosts from other studies. Orange – Asian primates (macaques). Blue – the reference sequence. Black – African primates. (TIF) [file ppat.1013240.s003.tif]
