# Supplementary material for: Phylogenetics and genomic variation of Hepatocystis isolated from shotgun sequencing of wild primate hosts
Source: PLoS Pathog. 2025 Jun 18;21(6):e1013240. doi: 10.1371/journal.ppat.1013240 (PMC12187015; doi:10.1371/journal.ppat.1013240)
Supplement: Attachment — Submitted filename: Response_to_reviewers_5March2025.docx [file ppat.1013240.s012.docx]

In light of the reviews (below this email), we would like to invite the resubmission of a significantly-revised version that takes into account the reviewers' comments.

The manuscript has been seen by four reviewers. Most found it interesting, but have made many suggestions that could improve the manuscript. One reviewer was quite negative. My own view is that the manuscript is potentially interesting, but I have two main comments. First, I think there is a need to modify the presentation and interpretation of the phylogenetic analysis, toning down the conclusions. (More details below.) Second, to overcome the comments of the negative reviewer, I think there should be much more emphasis on the results obtained from having mined the numerous nuclear genome sequences.

[1] A letter containing a detailed list of your responses to the review comments and a description of the changes you have made in the manuscript. Please note while forming your response, if your article is accepted, you may have the opportunity to make the peer review history publicly available. The record will include editor decision letters (with reviews) and your responses to reviewer comments. If eligible, we will contact you to opt in or out.

We thank the editor and all four reviewers for their comments, suggestions, and interest. As outlined in the responses below, we have substantially revised the manuscript to address the comments raised. Most notably, we have updated the phylogeny to include outgroups and modified the interpretation, and we have included additional information and analysis of sequence variation in the nuclear genome that is unique to this dataset.

Phylogenetic analyses:

Figure 3: The tree in part A, where only the new sequences plus one other (the “ref” sequence) are included, is redundant. The text (line 170) refers to the length of this central branch (0.032) as being meaningful, but this is misleading. The impression of a long central branch separating two clades disappears when other sequences are included (as shown in part B).

The tree in part B should be rooted in a more appropriate position. As one reviewer suggests, this could be achieved by including outgroup sequence(s). The closest outgroups are likely to be Hepatocystis from bats. The tree could also be presented more clearly. It looks like every sequence is version 1, and so the labels do not need to indicate this. You could perhaps preface each label with a 2-letter code for the genus.

Thank you for this important correction. We have removed figure 3A. We have added in an outgroup from an Asian fruit bat and rooted the tree. We have also relabeled the tips to give the genus and colored to highlight the sequences from our study.

The misleading tree in part A is used to suggest there are two species here (i.e., cHep is one, pHep plus “ref” is the second). Even after the inclusion of other sequences in part B, the text continues to refer (line 184) to the pHep sequences (plus others from other hosts) as “this species”, but it is not at all obvious how this species is demarcated. It would not seem to make much sense to suggest, from the tree in part B, that there are two species, where the second includes all sequences beyond cHep. But how many species are there here? (I think it impossible to decide.)

When the tree in part B is rooted in a more appropriate position, the clade of cHep sequences will seem less special; not very different from the clade of exclusively Piliocolobus-derived sequences near the foot of Figure 3B.

In addition, at line 292 it is suggested that pHep clustered with isolates from 6 different genera. However, in Figure 3B, the majority of the pHep sequences fall in a cluster with (only) other Papio-derived sequences, plus a single chimpanzee-derived sequence; as reviewer #3 suggests, the chimpanzee-derived sequence may not be a real infection. Only one pHep sequence falls outside this cluster; the larger cluster including that other pHep sequence has no sequences from Mandrillus, Miopithecus or Colobus. In fact, I can only see single sequences from each of these three genera in the tree, and they are all clearly more closely related to cHep than to pHep.

Finally, it seems ironic that the Introduction refers to the limitations of cytb phylogenetic analysis (line 89), but then the only phylogenetic analysis in the paper is cytb.

Thank you for these important points and corrections. We have revised the interpretation correspondingly, such that we now highlight that *Hepatocystis cytb* sequences sampled from each host genus cluster with near one another, but they are not in genera-specific clades. We also now highlight that we obtained identical *cytb* sequences from different individuals within the same host species, but not from different host species. We agree that a major novel aspect of our study is the ability to look at sequence variation across the rest of the genome. We have now extended our analysis of the genome-wide variation to look at population structure by computing principal components from the genotype likelihoods (now Figure 3). The first PC shows separation between the *Papio-*infecting and *Chlorocebus*-infecting isolates, and within Chlorocebus, there is clustering by host subspecies. This suggests that across the whole genome, the parasites are quite strongly structured by host. This is further discussed now in the paper’s discussion.

In conclusion, I suggest deletion of Figure 3A, rooting the tree in Figure 3B more appropriately, and a revision of the conclusions derived from this phylogeny.

Other comments:

Figure 1: not quite “by taxonomic group”. The boxes above place the OWMs in two groups based on geography. Taxonomy would cluster the first five species in Cercopithecini, and the next six species in Papionini. Genus names should be given (“P.” stands for three different names here.)

We have changed this to “by host genus and geography” and have also included genus names on the x-axis of the figure.

Line 240: It would help to explain that ACKR1 was previously known as DARC.

This has been added.

Lines 333-335: African apes have also been found to be infected with relatives of P. vivax and P. malariae (and probably the two P. ovale species). African monkeys (of several species) have been found infected with P. gonderi and P. mandrilli (previously referred to as DAJ-2004).

We have corrected these statements and added appropriate references.

Reviewer's Responses to Questions

**Part I - Summary**

Please use this section to discuss strengths/weaknesses of study, novelty/significance, general execution and scholarship.

Reviewer #1: In this important work, Haffener et al. expand the number of known genomic samples for the enigmatic parasitic genus Hepatocystis by two orders of magnitude, identifying reads captured as part of attempts to sequence primate genomes. In doing so they identify a new putative species of Hepatocystis, confined to certain host species, alongside other important insights. This is impactful work, which has been rigorously carried out and is well-described.

Back in 2018, this reviewer was involved in the first identification of a genome for Hepatocystis. This came about because in the process of sequencing a red colobous monkey, experimenters acquired sufficient reads that their final monkey assembly, deposited on NCBI, contained many contigs that were in fact from Hepatocystis. Puzzled by BLAST searches that reported that “monkey” genes were closely related to genes of the malaria parasite (<https://theo.io/blog/2018/04/23/how-i-stumbled-upon-a-novel-genome-for-a-malaria-like-parasite-of-apes/>), and we went on to characterise this genome in detail (<https://www.ncbi.nlm.nih.gov/pmc/articles/PMC7425995/>). That finding was the result of serendipity, and made possible by the large amount of Hepatocystis present in the sample.

Here, the authors instead take a targeted approach, they download the raw reads captured as part of a large number of primate genome sequencing efforts and look for samples containing Hepatocystis, by matching kmers to that initial genome (and also using other approaches). They survey 326 primate genomes and identify 30 Hepatocystis infections that have left significant genomic material, increasing the number of known genome-scale datasets of Hepatocystis by more than an order of magnitude. Although many (or all) samples have very low coverage, they are able to make use of these datasets in two main ways. Firstly, because mitochondrial DNA is much more abundant than nuclear DNA, they are able to accurately infer sequences for the samples' cytochrome B genes and infer a phylogeny. This phylogeny clusters by host species, with Chlorocebus-infecting Hepatocystis genomically distinct from those infecting baboons or colobus monkeys, the former representing a putative new species. Secondly, even though the samples' coverage is too low to be confident of the base at any particular position in a particular sample, the authors use a probabilistic approach to identify regions of the genome that show the most variation between samples. Their results here are plausible in terms of what we know about gene function in Plasmodium. Additionally, wherever the authors use new methodology they perform rigorous benchmarking using simulations based on malaria samples to demonstrate whether it has good power.

All of the claims made in the manuscript are well-demonstrated and carefully described. The identification that Hepatocystis sequences are widespread, of a new species with limited host range, of the lack of apparent species-specificity by geography, and of apparently highly variable genes in Hepatocystis are all important discoveries, as is the proof-of-concept that this work offers for its methodology.

- Theo Sanderson

Reviewer #2: The manuscript of Haffener et al. uses a bioinformatics approach to scour published genome sequencing projects of OWMs for sequences deriving from Hepatocystis parasites, followed by phylogenetics to investigate evolutionary relationships among putative Hep lineages and analyses of SNP variation to investigate genes putatively involved in adaptation. Overall, the results are potentially very interesting as little is known about the diversity and distribution of Hepatocystis parasites infecting OWMs. The text is well-written, and the methods are mostly clearly explained. The figures are nicely presented but some require additional clarification regarding what exactly is being shown. I think the core methodological approach employed is robust, although I was uncertain about some of the downstream methods used to arrive at their final number of ‘true’ infections, which I found to be perhaps overly complicated. A couple of key issues that I would like to see addressed before consideration for publication are outlined below.

Reviewer #3: This study provides crucial insights into the evolutionary history of parasites from the Hepatocystis genus, which are widely distributed among mammals, including rodents, bats, and primates. Nevertheless, the phylogenetic relationship between this genus and parasites of the Plasmodium genus remains a topic of ongoing debate. The findings presented in this study significantly enhance our understanding of these parasites. However, the impact of this work could be further amplified by refining a few minor aspects, which, in my view, would elevate the overall quality of this already excellent research.

Reviewer #4: The genus Hepatocystis remains understudied, largely due to difficulties to sample and monitor infections in wild hosts, incl. non-human primates (NHPs). The present study presents a dark-mining approach of publicly available shotgun sequence reads of NHP blood samples. Accordingly, the authors have not generated any original data, but merely assembled available data. They searched 326 samples from 17 NHP species and considered appr. 10% (30) samples infected with Hepatocystis, based on a cut off of 0.002% 'Hepatocytis-like' sequence reads. The vast majority of the samples (28) had reads < 0.1%; only two samples were a little higher. This can be attributed to the overall low parasite abundance due to lack of erythrocytic schizogony. What is the advancement over a similar study (Ref.3)? Perhaps another proof that dark-mining works, perhaps a slightly improved molecular phylogeny, and/or perhaps a first glimpse at the (expected) genomic variation. The latter is not surprising, and a finding of interest to a very limited research community. Fig.3 displays the molecular phylogeny of the few NHP Hepatocystis genome sequences, which, of course, vary a bit. No context to other Hepatocystis sequences can be provided, which is, of course, due to lack of reference data. The SNP density (Fig. 4) gives a first, albeit preliminary, impression of SNP frequencies. Sequence assemblies and SNP analysis are sound, but the study lacks any salient finding that advances our understanding of Hepatocystis-host interactions, parasite stage conversion or infection dynamics.

**Part II – Major Issues: Key Experiments Required for Acceptance**

Please use this section to detail the key new experiments or modifications of existing experiments that should be absolutely required to validate study conclusions.

Generally, there should be no more than 3 such required experiments or major modifications for a "Major Revision" recommendation. If more than 3 experiments are necessary to validate the study conclusions, then you are encouraged to recommend "Reject".

Reviewer #1: I believe the study's conclusions are already well justified.

Reviewer #2: General comments:

(1) The main pipeline uses Kraken2 to identify putative sequencing reads as deriving from Hepatocystis (or the primate host, or other Plasmodium) based on comparison of kmers (i.e., sequence identity). This seems like a sensible approach that I have no qualms about. However, I do wonder if the authors have sufficiently dealt with the potential problem of misidentification of sequences that in fact are from Plasmodium infections of OWMs, rather than Hepatocystis. Did the authors check the GC content of putative Hep reads? This should be distributed similarly to that seen for the reference Hepatocystis genome (i.e., around 20% GC, somewhat lower than most other Plasmodium species) and would be a relatively straightforward sanity check on the data. I understand that the authors ran their pipeline but searching for Plasmodium reads, rather than Hepatocystis, and came up with a null result (e.g., line 146), but it wasn’t mentioned if P. gonderi (as the only published genome of OWM-infecting Plasmodium species) was included in the reference set here. All this may not make a difference but it might be worth double-checking – my main concern would be if mis-identified Plasmodium reads have slipped through the net and are contributing to some of the ‘hypervariable’ regions identified downstream.

We appreciate the concern and have done some additional checks to address this. The mean GC content of reads classified as *Hepatocystis* is 24.7%, which is very similar to the GC content of *Hepatocystis* (e.g., in Aunin et al. figure 1A). We now include this in the results. The Kraken database does not include *P. gonderi* (we have now clarified which species are included in the methods), so to address the specific concern that *P. gonderi* sequences might nonetheless be present and contribute to hypervariable regions, we conducted competitive mapping. For each infected sample, we took the reads that had mapped to *Hepatocystis* and instead mapped them to a joint reference including both *Hepatocystis* and *P. gonderi* reference genomes. In all cases, we found a low percent of reads that mapped to *P. gonderi* instead of *Hepatocystis* (mean 1.5%, range 0.6%-3.2%), indicating *Hepatocystis-*specific mapping. Nevertheless, to ensure no *P. gonderi* sequences could be impacting the variant set, we removed the reads that preferentially mapped to *P. gonderi* and only used the *Hepatocystis-*mapped reads for variant calling, PCA, and SNP density analyses. These analyses have now been incorporated into the manuscript.

(2) Discussions about topological groupings (e.g. evidence for cHep and pHep putative species) based on the phylogenies shown in Fig. 3 are, at current, slightly meaningless because the trees are not rooted; monophyly of one set of taxa with respect to another is not possible without rooting the tree. This would be relatively easy to solve by including orthologous sequences from a range of other Plasmodium species and redrawing the tree, rooting as appropriate. This would have the added benefit of seeing relationships among putative Hep samples within the broader context of Plasmodium evolution. I strongly recommend additional sequences are added to at least Fig. 3B.

Thank you for this very important point. As also commented in response to the editor, we have removed figure 3A and have now added an outgroup bat-infecting Hepatocystis sequence to root the phylogeny. As noted, this changes the interpretation of the relationships among parasites from different hosts, and we have modified our statements and conclusions accordingly.

Reviewer #3: (No Response)

Reviewer #4: No experiments were performed in this study. The authors searched available sequence information and assembled the data. Accordingly, no validation, such as parasite microscopy, immunofluerescence stainings or qPCR analysis can be done.

**Part III – Minor Issues: Editorial and Data Presentation Modifications**

Please use this section for editorial suggestions as well as relatively minor modifications of existing data that would enhance clarity.

Reviewer #1: I stress again that the claims of the paper are already well-demonstrated. I do not see any of the below issues as essential to address for publication.

As a reader I initially expected the authors' approach to, as one small part "rediscover" the infection of the red colobus monkey that we had previously identified. In fact their survey does not include any colobus genomes. It could be useful to describe why - whether they were intentionally excluded as the only relevant ones had already been described (this is entirely fair, but is just not mentioned), or whether this is the result of some filtering.

We also initially thought this would be part of our analysis – but the raw read data from the red colobus monkey and thus infecting *Hepatocystis* are not publicly available. The RNAseq data analyzed in Aunin et al. is available, but we limited our analysis to DNA sequencing data only and so did not include it. We have added a note about this in the methods when discussing the data curation.

I support experimentation with new graphical approaches but I personally didn't find Fig 1. especially intuitive - I think I'd find a stacked bar chart with countries labelled more intuitive (but this is a matter of opinion).

Thank you for the feedback. We had trouble fitting so many country names onto a bar chart, and so have opted for this format.

Fig. 2: I'd suggest expanding the "IQR" label to indicate it refers to coverage

Thank you for this suggestion. In light of Reviewer 2 and 3’s comments, we have removed IQR from our analysis.

Fig. 3: It could be useful to include an outgroup, such as a malaria cytb in some figure to put the relationships into context?

Indeed, as the other reviewers and editor pointed out this was an error that we have now corrected. We have updated Figure 3, the results and interpretation accordingly.

The authors simply describe the mean coverage of their samples as being very low, leaving open the possibility that they might have number of high coverage samples (though glancing at the BAM files this does not seem to be the case) - it would be great to give a bit more detail on the distribution of coverage, providing some sense of for example whether there are further sequences of sufficient coverage to allow assembly. Given that the colobus discovery was so accidental, I had expected that a targeted survey might discover more high coverage genomes, so if that was not the case that result seems worth reporting. I'm also interested in the extent to which cHep samples could be pooled to give a reasonable assembly. A supplementary table including coverage for each sample could be nice.

Abstract: "low coverage whole genome sequence (lcWGS) data from 326 wild non-human primates (NHPs) in 17 genera." This suggests that all samples the authors analysed had low (host) coverage. Is that the case? If so is that because all wild-primate genomes are thus, rather than due to filtering specifically for low coverage sequences?

Thank you for pointing this out. We did not do any filtering or focus on low coverage primate sequencing, but much of the primate data out there is low coverage. The samples where we identified *Hepatocystis* all happen to have relatively low coverage (mean host genome coverage of 1.6X for *Papio* and 3.5X for *Chlorocebus*). The colobus dataset in which the initial discovery genome assembly could be done were from a colobus reference genome project, where the host individual was sequenced to 100x coverage. We have updated the abstract to refer only to WGS to make this clearer and added additional discussion of the range of host coverage. We have added a supplementary table (now Supplementary table 2) with individual-level metrics including the mean coverage of each infected sample – both for the primate and *Hepatocystis* genomes. The mtDNA and nuclear coverage for *Hepatocystis* is now also shown graphically in Figure 2A.

With your finding of hypervariability in the HEP families: I can imagine a scenario in which highly duplicated gene families could complicate mapping and generate spurious variability (but of course a finding of genuine variability is extremely plausible in such cases do, as with the var genes). I guess that's just worth bearing in mind. I'm not requesting a change to the text.

This is a possibility. We have now removed sites with very high depth from the SNP density analysis and still recover hypervariability in HEP genes. We have added a sentence about this into the discussion. We do observe a weak correlation between sequence depth and SNP density (not shown), but we also would expect this as we have more power to identify SNPs when coverage happens to be higher.

Sometimes you use "vHep" and I'm unclear what is meant there (sometimes it is contrasted with pHep and sometimes cHep)

Thank you for catching this. These are typos and have been corrected to remove reference to “vHep”.

You mention Papio and you mention baboons. It might be nice to draw out the synonymous nature of these to the reader sometime to help the ignorant (like me)

We have added the common names into the text.

In my view, all researchers should try to share their code as much as possible to help others to understand the details of their methods. To the extent that the authors have code for their analyses it could be useful if they could share this on Github. The code need not be polished.

Reviewer #2: Specific comments:

Line 133. “For further evaluation, reads from all Papio and Chlorocebus samples were mapped to a joint primate and Hepatocystis reference genome” I think other Plasmodium genomes should be included in this mapping step, as well as the Hepatocystis reference and other primate genomes. The inclusion of Plasmodium sequences would act as ‘decoys’ (similar to mapping strategies in RNA-seq analyses) to filter potential true Plasmodium reads from the final set.

Instead of mapping to multiple references and using Plasmodium sequences as decoys, we opted for a two-step approach where we used Kraken first to help distinguish between many related parasite species. Our concern is that if we map to a reference including both *Hepatocystis* and *Plasmodium* genomes, many reads will match all of them to some degree (be “multi-mapped”) and thus have a low mapping quality. Instead, we first compared reads against a large number of *Plasmodium* reference genomes using Kraken, which uses unique kmers to distinguish between similar reference genomes. Once we were confident in which species Kraken was identifying, we then mapped to the joint reference. We have added a discussion of this into the manuscript.

Line 141. It would be helpful if more information was provided in terms of the results from each ‘infected’ dataset, in terms of recovery of putative Hepatocystis sequences. Proportion and length of Hep reference genome with reads mapping to it? Number of Hep genes covered, fully/partially? Any BUSCO genes covered? Average coverage is fine but it would be good to have additional details of the actual results here to aid understanding of these initial data.

Thank you for this suggestion. We have now added Figure 2A and a supplementary table that shows the mean depth of the *Hepatocystis* genome, and Supplementary Figure 2 showing the proportion of sites with each coverage for each sample. Unfortunately, infected samples have low host genome coverage and thus do not have high coverage of the nuclear genome (range 0.1-0.76). Coverage of genes was essentially the same as overall coverage of the nuclear genome.

Line 161. Did you attempt to assemble the putative Hep reads into contigs, other than for cytB?

Unfortunately, coverage is too low across all samples to do this. The maximum nuclear Hepatocystis coverage for an individual sample is 0.76X. Even combining coverage across individuals, the mean coverage is 6.3X for Chlorocebus and 4.6X for Papio, which is lower than needed for confident *de novo* assembly.

Line 145. “Although we applied the same pipeline to look for Plasmodium, we found no clear infections supported by multiple predictors.” Could you expand on what exactly was done here? Results of these analyses could potentially answer my point above.

We have added some more details on this. Figure S1B now shows a representative example of what the % reads classified as *Plasmodium* looked like, shown for *P. cynomolgi.* There were a handful of samples that had somewhat higher % reads classified than others for a particular *Plasmodium* species, or combinations of host+parasite where the % reads seemed to always be more elevated compared with the French negative control samples. Upon mapping to a joint reference, in all cases we recovered essentially little to no mtDNA and coverage in very limited parts of the nuclear genome, suggesting it resulted from errors in the reference genome, repetitive sequences, or other technical issues.

Line 136. “K-means clustering using the ratio of mtDNA to nuclear coverage, a measure of coverage uniformity (interquartile range), and the percent of reads classified revealed two out of three clusters that corresponded to elevated values across the three predictors.” I don’t fully understand this approach. What is the justification for choosing these 3 ‘predictors’ (ratio mt/nuclear, IQR coverage and % reads classified by Kraken) over others? It seems unnecessarily complicated – why not simply choose reads which preferentially map to the Hepatocystis reference genome, over primate and other Plasmodium decoys?

Thank you for this feedback. In light of this and reviewer 3’s comments, we have decided to simplify the approach. However, we believe that choosing reads that preferentially map to *Hepatocystis* over *Plasmodium* decoys is problematic for two reasons (as also explained above). First, we will lose true Hepatocystis reads that also map fairly well to Plasmodium genomes. Second, the problem with just looking at the % reads classified by Kraken is that there is a level of ‘noise’ whereby any sequence dataset will have some % reads that are classified as *Hepatocystis*. This can result from numerous possible sources – e.g., sequencing errors that happen to create kmers that match *Hepatocystis*, errors in the reference genome creating sequence matches to the experimental sequences (e.g., contamination common to both the reference genome and sample sequences).

Fig. 2A. (i) There is quite a lot of blank space in this plot, is it necessary to show all the negative results? It’s also not clear from the plot itself that the X-axis of the bar plots represents the 326 samples, which, unlabelled and grouped by host genus, make it appear as if there is equal sampling across all 6 genera. (ii) Why choose 0.002% as the cut-off? Is this the 6 SDs difference compared to the French HGDP ‘negative control’ mentioned in the Methods? Please clarify.

We hoped this plot would convey the breadth of samples analyzed, and that most were negative even. However, we appreciate this feedback and we have opted to move this figure to the supplement. Instead, Figure 2 now shows the mtDNA and nuclear coverage for each sample in Chlorocebus and Papio, colored by whether they had more than 0.002% of reads classified. Yes, 0.002% was chosen as a guide as this corresponds to more than 6SDs above what was observed in the French “negative control” population. We have clarified this in the figure legend. We have now moved the previous figure to the supplement (Figure S1), where we show it for *Hepatocystis* and for *P. cynomolgi* as an example for *Plasmodium.*

Fig. 2B. (i) What are the points? Are they the 42 samples with > 0.002% Hep reads identified in part A? Please clarify. (ii) It would be helpful to briefly remind readers what IQR is and how it is calculated when defining the Y-axis. (iii) Also, the colour of cluster B vs cluster C is very hard for me to differentiate on the plot.

As suggested, we have simplified this inference and so no longer include IQR, the clustering, or Fig. 2B.

Figure 3. What is the X-axis? Genomic position of the Hep reference genome? Please clarify on the figure.

Yes, this is position along the genome, with contigs ordered by size. We have added an axis label and now explain this in the legend.

Line 192. It is unclear exactly how the mapping was performed during SNP calling, even in the Methods (see later comment). Either way it might be helpful to have a quick recap of how these variants were generated written here.

The mapping was performed prior to SNP calling by mapping reads to a joint *Hepatocystis* – primate host reference genome with bwa mem. SNP calling was then performed from the reads mapped to Hepatocystis contigs using ANGSD. We have clarified this in the methods and added more details about how the SNP calling was done.

Line 357. Did the SRA search not flag the sequencing project known to contain the Hepatocystis reference genome previously analysed by Aunin et al. (2020)? It doesn’t seem to be mentioned in any of the results presented, was it manually removed at a later stage? It might serve as a useful positive control for some of the later analyses.

That is correct. The raw read data from the red colobus monkey and thus infecting *Hepatocystis* are not publicly available. This individual was part of a reference genome project for which the assembled contigs are available, but not the raw read data. We now mention this explicitly.

Line 369. Which Plasmodium species were included in the Kraken2 database? Was the OWM-infecting species P. gonderi included? If not, it might be useful to include this species to double-check if any reads are flagged as Plasmodium.

No, *P. gonderi* was not included in the standard Kraken2 protozoa genomes at the time we constructed our database. We have now clarified which species are included in the methods. To address the specific concern that *P. gonderi* might be present, we conducted competitive mapping with *Hepatocystis and P. gonderi* references and removed reads that mapped to *P. gonderi* (see also response to reviewer #2 major comment #1 above)

Line 413. I don’t fully understand the approach for identifying samples with putative Hep infection, which seems overly complicated. The first 2 indicators are reasonable enough, but what is the justification for the IQR metric? Unless I am mistaken a higher IQR value corresponds to more variable coverage across the Hep reference genome – why would this correspond with ‘true’ infection? In addition, for the k-means clustering part, why k = 3? Is there a reason for choosing this value? I do wonder if this part could be simplified – why not just include all samples with > 0.002% Hep reads and a reasonably complete cytB gene? The clustering analyses seems to have removed 12 samples (n reduces from 42 to 30, line 138); you could reasonably include all 42 samples with the caveat that some may have limited evidence, but presence of a cytB (or other marker) clustering with other Hep sequences is more convincing to me.

We aimed to capture an aspect of the evenness of distribution of coverage along the nuclear genome with IQR, as we observed that samples sometimes showed nuclear coverage in only a very limited section of the genome. However, this is not an ideal metric and we have instead now looked more explicitly at the relationship between the proportion of the genome at each coverage (see figure S2). We have also simplified our inference to use only the % reads classified and nuclear and mtDNA coverage. We do not only consider cytB because (1) we would need to decide what “reasonably complete” corresponds to, and (2) we want to analyze genetic variation in the nuclear genome even when coverage is low, as this is a unique aspect of our dataset. We may still miss other samples that are, in fact, infected with *Hepatocystis*, but for which the sequence data is more limited (e.g., as can be seen in Fig. 2A, particularly for *Papio*). Due to the low coverage of these samples, there is a limit to the sensitivity and it becomes difficult at very low coverage to distinguish true infections from noise in read mapping and classification.

Line 438. How exactly was mapping performed prior to SNP calling? Were reads from different samples assigned unique read group IDs and all mapped together, or within pHep and cHep subgroups, or where individual samples mapped separately? Please provide a bit more detail.

The reads from each sample are in separate fastq files (as downloaded from SRA) and are each mapped separately with bwa mem. We now state this more explicitly. Mapping was done per individual sample. Variant calling was done for all samples together, as well as separately for cHep and pHep. We have now included more detailed description in the methods.

S2 Fig. What is shown on the X-axes? The 329 FastQ samples? Please clarify. Also, why group samples by cluster? This seems kind of circular, since some of these metrics are used to define the clusters, but also biologically meaningless, since cluster ID is based on technical properties of the sampling strategy (i.e. probably influenced by things like type of extraction kit used, blood sample protocol, sequencing depth, primers used, amplification protocol (or not), etc…) rather than anything to do with the host or parasite itself.

We have removed this figure as we are no longer using this clustering approach. We had intended for Fig. S2 to show that there are samples with low coverage that are more challenging to classify, but we now show the coverage explicitly in Figure 2A.

Minor comments:

- Line 149. “Samples are grouped by >host< genus.”

This has been added.

- Line 154. “pHep” and “cHep” need to be defined.

We now define these at first usage.

- Line 291. “isolates” should be unitalicized.

This has been corrected.

- Line 389. What is the “French HGDP population”? Please define/clarify.

We now clarify that this refers to “French individuals in the Human Genome Diversity Project”.

- Line 408. How was the alignment performed? What tools/parameters?

This was done with bwa mem. The details of alignment have now been added into the methods.

- Line 418. Presumably this was done for each sample separately? Please clarify.

Yes, read mapping and coverage calculations were done for reach sample separately. This is now stated explicitly.

- Lines 424 and 436. Please specify which model was used to construct the distance matrix for tree building. Was model testing implemented? A more recently published phylogenetics tool like IQ-TREE is able to run model testing

Thank you for this suggestion. We have now used IQ-TREE and run model testing. The best model was GTR+F+R2 and we now include this information in the methods section.

Reviewer #3: Minors corrections

Introduction

The text is well-structured, moving logically from background information to the study's objectives.

The use of phylogenetic context sets the stage for understanding the significance of Hepatocystis in relation to Plasmodium.

The authors are thorough in referencing previous studies, providing a solid foundation for the research.

It effectively highlights the need for genomic data to better understand Hepatocystis, indicating a gap in current knowledge that this study aims to fill

Howover, to improve this introduction, it could benefit from a clearer research question, a more focused explanation of the study's significance, and a brief overview of the methodology. Additionally, balancing the background information with a stronger emphasis on the novelty and broader implications of the findings would make the introduction more compelling.

For examples, the authors could rewritte some sentences which very long

authors could simplify sentences to make them less complex

Thank you for the suggestions to improve the readability of the manuscript. We have edited the end of the introduction to highlight the research question, novelty and broader implications particularly focusing on the ability to access genetic variation across the genome rather than limited to a single locus. We have also edited the document to simplify and clarify longer sentences.

1. Line 58-60 remove the apicomplexan and use only Hepatocystis parasites or genus.

This has been removed.

2. Line 60 delete the most and consider only Notably and rewrite this senence. I suggest you for example: “Notably, Hepatocystis parasites do not undergo asexual replication in red blood cells, which is responsible for the symptoms of malaria, and are transmitted by midges of the Culicoides genus rather than by Anopheles mosquitoes

This has been rephrased.

3. Line62-65 this sentces is very long, could rewrit that, please

This sentence has now been broken into shorter sentences.

4. Would the lack of data provided by studies of Hepatocystis in rodents and bats not constitute a lack of appreciation of the positioning or analysis?

Our apologies, it is not clear to us what this comment refers to.

5. Please rewrite this sentence is not clear, line 85-88

This sentence has been rephrased.

6. Is there really any proof that chimpanzees are infected with Hepatocysitis? Given that chimpanzees feed on small monkeys and that this has been observed in the faeces, wouldn't this be the consequence of this? Also, is there a study using blood that shows this?

We agree with this skepticism - the study referenced reports only one chimpanzee blood sample from which *Hepatocystis* sequence was obtained, and this is the only one we are aware of. We have modified the text to indicate that the chimpanzee sequences are largely from fecal samples and could reflect other primate hosts instead.

7. Line 96 “spp” is not italicised, change it throughout the manuscript,

This has been corrected throughout.

8. Line 107, “spp” spp is not italicised, change it throughout the manuscript

This has been corrected throughout.

Results

Curation

1. I think that the clarification of the purpose of databases mentioned here, enhancing the relevance of the data to the study

We have added that the NCBI SRA database is a repository for raw, shotgun DNA sequence data and metadata on sampling location and source material.

2. There is some redundancy in the percentages provided for the sample distribution. For example, "Of the remaining samples, 12% were collected in the Caribbean Islands and the other 12% were collected in Asia" can be streamlined

This has been streamlined.

3. While the text mentions the SRA database and BioProject, it could be more explicit about what kind of data or information these databases provide to reinforce their relevance to the study.

We have added more detail in the methods about the type of data and metadata, and why this is appropriate for this study (also see response to #1 above).

4. Line 125: The definition of the term 'OWM' is superfluous, given that it has already been provided in the introduction. There is no reason for it to be repeated here.

Since readers may reference the figures separately from the text, we have kept the definition in the figure legend but ensured there were no other repeated definitions.

Hepatocystis infections in African Old World Monkeys

1. This section of the text is characterised by a high level of technical vocabulary and complex sentence structures, which may prove challenging for readers with limited familiarity with the subject matter. The text could be made more accessible to a wider audience by simplifying some sentences and breaking down complex ideas into more digestible parts.

We have edited this section into shorter sentences.

2. For example, it would be beneficial to rewrite lines 129-131, as they currently contain multiple ideas that could be more effectively conveyed through the use of simpler sentences.

This sentence has been rephrased.

3. Lines 136–138 comprise a dense and complex sentence, combining multiple metrics and results into a single unit of information. The text could be made more accessible by breaking it down into smaller sentences or simplifying it.

This approach has been changed and so this section has been rewritten.

4. For instance, lines 143-146 could be rewritten as follows: "Additional cases with elevated values for one or two predictors may also represent true infections, especially those with lower overall coverage."

This has been rewritten in light of the changes to our approach.

5. Please clarify why the authors do not use spp when discussing Hepatocystis or Hepatocystis parasites. Do the authors consider that this is a single species of Hepatocystis, or are they discussing the genus hepatocystis?

Thank you for this point. We do not claim whether there is one or more than one *Hepatocystis* species represented here. We have added this point to the discussion and refer to *Hepatocystis* spp.

Genomic variation in Hepatocystis

The present section comprises a number of lengthy sentences that may prove confusing to the reader. It is therefore recommended that the authors shorten some of them.

For example "We used precision recall curves to determine a threshold for considering windows of high SNP density (S3 Fig). Using 1kb bins in the 30X data with a SNP density greater than 2 standard deviations from the mean as the truth set, both 1X and 0.5X datasets had high precision (100% for the top 10 bins and >96% for the top 50 bins) and overlapped genes known to be hypervariable in P. falciparum (S3 Table).

This has been edited to clarify.

Discussion

The discussion is well written, although it does exhibit some shortcomings. However, these do not detract from the excellent quality of the work as a whole. Nevertheless, addressing these issues would enable the discussion to be presented in a more comprehensive, accurate and relevant manner to a broader scientific audience.

1. The discussion makes general observations without providing a detailed analysis of the genetic differences between the newly identified Hepatocystis species and other known species. This lack of detail makes it challenging to ascertain the significance of the findings.

The nature of this data is that we cannot perform a detailed sequence comparison as most parts of the genome do not have coverage in all samples. We hope that adding in the PCA analysis based on genotype likelihoods helps show some more insight into population structure.

2. The functional implications of the genetic variations observed in Hepatocystis in comparison to Plasmodium have not been sufficiently explored. The discussion does not elucidate how these differences might affect the parasite's biology or its interaction with hosts. It would be beneficial for the authors to develop this aspect further in their discussion.

We elaborate on some hypotheses in the discussion based on the genes appearing as hypervariable, but we can only speculate at this point. Nonetheless, we feel that the first look at genomic variation still offers an exciting opportunity to do so.

3. The discussion provides a cursory overview of the co-evolutionary dynamics between Hepatocystis and its NHP hosts, yet lacks a comprehensive analysis. This leaves a gap in our understanding of the evolutionary dynamics at play.

We have added to the discussion, in particular that a deeper understanding of the co-evolutionary dynamics will require further data. Our study shows that this will be possible with future samples from primate host blood.

4. The following assumptions are made about host specificity: The supposition that a singular generalist Hepatocystis species may infect a range of diverse African NHPs is posited without substantial corroboration. This weakens the argument and fails to consider the potential for more complex host-parasite relationships.

We have rephrased this as a more general statement: “Phylogenetic trees of *Hepatocystis* *cytb* sequences isolated from different species of African OWMs and chimpanzees exhibit limited geographic clustering or host-specificity.”

5. It is notable that the analysis relies heavily on a single genome sequence, which may limit the generalisability of the findings. Furthermore, the discussion does not adequately consider how additional genomic data might alter current interpretations. It would be beneficial for the authors to take this into account for a more comprehensive assessment.

We were not sure if this refers to the use of a single reference genome, as a strength of our analysis is inclusion of 37 different *Hepatocystis* genomes. In the discussion we mention how future genomic data may lead to additional insights.

6. Additionally, the limitations of the methods used, such as potential biases in the taxonomic classification tool (Kraken2) or challenges in mapping reads, are not sufficiently discussed. This could lead to an overestimation of the results' reliability.

We have added further discussion of some of the challenges and limitations, both in justifying our choice of methods and in the discussion section.

Reviewer #4: Due to the lack of original data the authors maximized their display items. Fig. 1 (the NHP samples) will typically be supplemental data, even in very specialized journals. Similarly, Fig. 2, which contains no central information and simply describes the set of 30 samples with low Hepatocystis sequence reads.

We appreciate this comment, but we felt that Figure 1, which illustrates the dataset, is helpful to the reader as a main figure. We have now added a supplemental table with details on all samples. We have moved Figure 2 to the supplement and replaced it with a more informative figure showing individual level coverage metrics.
