# Supplementary material for: Phylogenetics and genomic variation of Hepatocystis isolated from shotgun sequencing of wild primate hosts
Source: PLoS Pathog. 2025 Jun 18;21(6):e1013240. doi: 10.1371/journal.ppat.1013240 (PMC12187015; doi:10.1371/journal.ppat.1013240)
Supplement: Attachment — Submitted filename: Response_to_reviewers_8April2025.docx [file ppat.1013240.s013.docx]

**Additional Editor Comments:**

Dear Dr. Leffler:

Thank you for your revised manuscript. I am happy with the manner in which you have dealt with the various points raised by the reviewers.

However, in reading through this revised version, I was struck by a few points, particularly in regard to figures 3 and 4.

Thank you for these points, which we have addressed in the resubmitted manuscript and respond to point by point below.

Figure 3:

1. As suggested, you have added an outgroup to the phylogenetic analysis (Figure 3). Looking at the trees in Ref.9 (Schaer et al., 2017), it seems that the sequence from Pteropus (FJ168565) would be less distant from the primate-derived sequences, than the sequence from Cynopterus (MW366842) that you have used. Perhaps it would be better to use this other, closer outgroup?

We have repeated the phylogenetic analysis with *Pteropus* as an outgroup. This tree is now the one shown in Figure 3. The trees are very similar and have not changed the interpretation in the text.

2. The second sequence in the tree is labelled Pan. OL691976. But the GenBank entry OL691976 is described as being from a human sample.

Thank you, we missed that. We have updated this as *Homo* and added this finding to the text when describing *Hepatocystis* sequences recovered from chimpanzee fecal samples.

3. Sequences 7 and 8 in the tree lack accession numbers.

Thank you for pointing this out. These have been added in the figure (JF923757 and JF923759).

4. The legend to Figure 3 mentions an alignment length of 727 bp. However, the analysis includes many sequences from the Ayouba et al. (2014) paper, some of which are much shorter that this. For example, JQ070897 is only 518 bp; i.e., nearly 30% of the alignment length is missing. It would convey more accurate information if you indicated the number of sites present in all sequences in the alignment.

We have now added this information in the figure legend. That is correct, some sequences are shorter and somewhat different regions have been sequenced across studies: the minimum sequence length is 494 bp, and the number of sites present in all sequences is 287 bp. Given this is quite a bit smaller than the full alignment length, we have also built a phylogenetic tree from the 287 bp alignment and added it as supplementary figure 3. The overall relationships among sequences, particularly those from *Chlorocebus* and *Papio* that we present in the context of the published sequences, is consistent. We have also made the multiple sequence alignments available on Zenodo with the other materials for this manuscript.

Figure 4:

5. You have removed Fig.2A from the first version of the manuscript. You have inserted Figure 4, which potentially suffers from the same problem that Fig.2A had, that is the lack of samples placed at intermediate positions in the Hepatocystis tree. Thus, again your conclusions about the evidence for structure by host species (e.g., at line 35-36 in the Abstract) could be biased by the particular samples included in the analysis.

In the case of Figure 4, you could not include many samples, because most do not have nuclear genome coverage. However, it seems that you could (should) include the reference sample from the Piliocolobus genome in this analysis.

First, we would like to clarify that in Figure 4, all 37 samples are included. Although nuclear coverage is low and variable across samples, all do have some nuclear coverage and the approach we used accounts for this by working with genotype uncertainty (likelihoods). By combining information across the whole genome, genetic similarity among individuals is summarized along the principal components that explain the most variance across the thousands of genetic variants. We agree that this does only show relationships among the samples that are included in our analysis, leaving open how these are related to any others. Since there are no other such nuclear genomic datasets for primate *Hepatocystis*, we cannot include any others in this analysis. Nevertheless, this provides new and valuable information about the genetic similarities among these samples, akin to common analyses of population structure in other taxa (see, for example, analysis of low-coverage chimpanzee genomic data from different subspecies and sampling locations in Fig. 1F and 1G in this paper: <https://www.sciencedirect.com/science/article/pii/S2666979X22000623>).

There could be a way to include the reference sample from *Piliocolobus*, but there are several issues with this: (1) the raw data for this sample is not publicly available, (2) including a single individual from this host species would be difficult to interpret, as we would not have a comparison of within vs. between host species variation, and (3) this individual is the reference genome against which we have called SNPs. Thus we have not added in the reference sample. However, we have modified the text to emphasize that all 37 samples have been included and to clarify the interpretation and its limitations. As there is no outgroup, this also does not indicate any ancestral relationships.

Other minor comments:

6. L.313-314. Would it not be true to say that “all Chlorocebus species” were considered as Cercopithecus?

We have modified this sentence to state this more generally: “*Chlorocebus* were formerly considered to be in the genus *Cercopithecus”.*

7. L.337. “P.” should be “C.” Also, I note that Svardal et al. (2017; your Ref. 42) did not show hilgerti and pygerythrus as closer to each other than to cynosuros; I wonder why you consider these two (only) as subspecies?

We corrected the typo on line 337. As for how to refer to *hilgerti*, *pygerythrus*, and *cynosuros*, there does not seem to be a definitive answer. We chose to refer to these using the ITIS taxon names that were listed in the Svardal paper (their Supplementary data 1) as this also helps keep the link between the samples. In the Svardal paper itself, they do not recommend any change in what the species or subspecies names should be. In the discussion, they write: “There has been considerable debate about vervet taxonomy, both concerning the taxonomic levels of different groups (species or subspecies) and relationships between groups. While taxonomic assignment can reflect a variety of morphological, genetic and behavioral information, our results suggest that—despite evidence for substantial genetic exchange—***Chlorocebus* includes both genetically well-separated taxa (*sabaeus*, *aethiops*, *tantalus* and *pygerythrus*) and more closely related groups (*pygerythrus*, *cynosuros* and *hilgerti*). The latter groups would naturally fall at a taxonomic level below the former.”** We read this to suggest that they do not recommend raising *pygerythrus* and *hilgerti* to species level, yet neither do they offer a way to incorporate *cynosuros* at the subspecies level. When we checked the current ITIS taxonomy, it actually appears that *hilgerti* is now listed as “*Chlorocebus aethiops hilgerti*” which does not make sense with the genomic data. We have now added some additional discussion about this (lines 365-374).

I hope you can address these points.

Yours sincerely,

Paul Sharp


**Journal Requirements:**

1) Please include the affiliation of the author (Ellen M Leffler) in the online submission form.

We have added this affiliation to the best of our ability to find the correct location to do so on the online submission form.

2) Please include the source details of the map found in (Figure 2B) in the legend of the figure.

We have added this to the figure legend.
 
